# Copper Slag Cathodes for Eco-Friendly Hydrogen Generation: Corrosion and Electrochemical Insights for Saline Water Splitting

**DOI:** 10.3390/ma18133092

**Published:** 2025-06-30

**Authors:** Susana I. Leiva-Guajardo, Manuel Fuentes Maya, Luis Cáceres, Víctor M. Jimenez-Arevalo, Álvaro Soliz, Norman Toro, José Ángel Cobos Murcia, Victor E. Reyes Cruz, Mauricio Morel, Edward Fuentealba, Felipe M. Galleguillos Madrid

**Affiliations:** 1 Centro de Desarrollo Energético Antofagasta, Universidad de Antofagasta, Antofagasta 1240000, Chile; susana.leiva.guajardo@ua.cl (S.I.L.-G.); edward.fuentealba@uantof.cl (E.F.); 2 Departamento de Ingeniería Mecánica, Universidad de Tarapacá, Arica 1100000, Chile; 3 Departamento de Ingeniería Química y Procesos de Minerales, Universidad de Antofagasta, Avenida Universidad de Antofagasta 02800, Antofagasta 1271155, Chile; luis.caceres@uantof.cl; 4 Departamento de Química de los Materiales, Facultad de Química y Biología, Universidad de Santiago de Chile, Av. Libertador B. O’Higgins 3363, Santiago 9170022, Chile; victor.jimenez@usach.cl; 5 Departamento de Ingeniería en Metalurgia, Universidad de Atacama, Av. Copayapú 485, Copiapó 1530000, Chile; alvaro.soliz@uda.cl; 6 Facultad de Ingeniería y Arquitectura, Universidad Arturo Prat, Iquique 1100000, Chile; notoro@unap.cl; 7 Instituto de Ciencias Básicas e Ingeniería, Universidad Autónoma del Estado de Hidalgo, Carr. Pachuca—Tulancingo, Mineral de la Reforma 42184, Hidalgo, Mexico; jose_cobos@uaeh.edu.mx (J.Á.C.M.); profe_4948@uaeh.edu.mx (V.E.R.C.); 8 Departamento de Química y Biología, Universidad de Atacama, Av. Copayapú 485, Copiapó 1530000, Chile; mauricio.morel@uda.cl

**Keywords:** copper slag, corrosion, physicochemical characteristic, electrochemical characteristic, sustainable hydrogen technologies

## Abstract

The increasing demand for sustainable energy and clean water has prompted the exploration of alternative solutions to reduce reliance on fossil fuels. In this context, hydrogen production through water electrolysis powered by solar energy presents a promising pathway toward a zero-carbon footprint. This study investigates the potential of copper slag, an abundant industrial waste, as a low-cost electrocatalyst for the hydrogen evolution reaction (HER) in contact with saline water such as 0.5 M NaCl and seawater, comparing the electrochemical response when in contact with geothermal water from El Tatio (Atacama Desert). The physicochemical characterisation of copper slag was performed using XRD, Raman, and SEM-EDS to determine its surface properties. Electrochemical evaluations were conducted in 0.5 M NaCl and natural seawater using polarisation techniques to assess the corrosion behaviour and catalytic efficiency of the copper slag electrodes. The results indicate that copper slag exhibits high stability and promising HER kinetics, particularly in seawater, where its mesoporous structure facilitates efficient charge transfer processes. The key novelty of this manuscript lies in the direct revalorisation of untreated copper slag as a functional electrode for HER in real seawater and geothermal water, avoiding the use of expensive noble metals and aligning with circular economy principles. This innovative combination of recycled material and natural saline electrolyte enhances both the technical and economic viability of electrolysis, while reducing environmental impact and promoting green hydrogen production in coastal regions with high solar potential. This research contributes to the value of industrial waste, offering a viable pathway for advancing sustainable hydrogen technologies in real-world environments.

## 1. Introduction

Population growth and industrial activity drive an increasing demand for sustainable energy and clean water, leading to a greater dependence on fossil fuels. This reliance depletes the planet’s finite resources and exacerbates environmental impacts [1]. The decarbonisation of the energy sector has underscored the need for alternative and clean solutions [2]. A promising approach is hydrogen production using solar energy, known as solar hydrogen (H_2_). This is generated through water electrolysis powered by electricity from sunlight, making it sustainable, As solar energy is clean, abundant, and globally accessible. H_2_ production is modular, scalable, and adaptable to varying demands. Its use represents an effective strategy for reducing CO_2_ emissions [3,4,5].

Saline water electrolysis is a promising alternative for converting solar energy into hydrogen (H_2_), contributing to the energy crisis. However, there is a crucial need to develop efficient, durable, and cost-effective electrocatalytic materials for the hydrogen evolution reaction (HER) [6]. Enhancing the design of these materials has become a key objective. In this context, copper slag, a by-product of the copper smelting process, is increasingly recognised as a valuable resource for emerging electrochemical technologies. Globally, approximately 30 million tonnes of copper slag are produced annually, with Chile contributing around 4.5 million tonnes [7]. Traditionally considered industrial waste, copper slag contains valuable metal oxides such as hematite (Fe_2_O_3_), covellite (CuS), and magnetite (Fe_3_O_4_), which exhibit synergistic electrocatalytic properties. Its abundance in copper-producing countries such as Chile makes it an attractive alternative to conventional materials [8,9,10,11,12,13,14,15].

An analysis of world metal consumption statistics reveals that copper ranks third globally, surpassed only by steel and aluminium. The primary geological genesis of copper, accounting for approximately 90% of global extraction, is associated with sulphide deposits (CuS-FeS) [16,17]. To obtain copper, the ore must be concentrated and separated from gangue through a high-temperature pyrometallurgical process. In this process, Cu_2_S acts as a reducing agent, while oxygen from the air oxidises iron into iron oxide. This oxide then reacts with silica and lime, forming a silicate that is removed along with the slag. As a result, a significant amount of waste is generated, typically containing 30–40 wt% iron in oxide form, 25–40 wt% silica (mainly in the form of iron silicates), less than 11–12 wt% alumina and calcium oxides, 4–5 wt% other oxides (MgO, MnO, TiO_2_, P_2_O_5_, Na_2_O, K_2_O), 1–1.5 wt% sulphur, and approximately 1–1.5 wt% of copper [17,18]. Studies suggest that copper slag can generate hydrogen at rates of up to 0.113 μmol/g·h under photocatalytic conditions [19]. The integration of seawater as an electrolyte in hydrogen production further enhances the sustainability of the process [20]. With an extensive coastline of 6435 km, Chile presents significant potential for hydrogen production in coastal areas with abundant solar energy. A prime example is the Atacama Desert (Chile), which stretches from Arica to La Serena, covering nearly 1600 km in length and up to 180 km in width. Known for its exceptionally high solar radiation levels, the Atacama Desert is a strategic region for green hydrogen projects. However, seawater contains dissolved salts and minerals that can affect hydrogen production efficiency and accelerate corrosion in critical electrochemical system components [21,22,23].

Despite these challenges, seawater has demonstrated potential as an electrolyte, particularly when combined with copper slag. Its mesoporous structure and high stability in saline environments allow it to resist corrosion while facilitating the electron transfer processes essential for hydrogen evolution. Several studies have explored hydrogen production using copper slag as a Fenton-type catalyst in advanced oxidation processes for water treatment. The presence of Fe^2+^ and Fe^3+^ oxidation states in copper slag enhances the generation of hydroxyl radicals, which play a crucial role in breaking down contaminants in wastewater [19,20,24]. The integration of copper slag into electrocatalytic systems for hydrogen production and water treatment addresses key environmental challenges while offering numerous sustainable benefits. Its use aligns with strategic objectives such as (i) waste valorisation, (ii) transforming industrial by-products into valuable resources, (iii) reducing slag accumulation, and (iv) generating added value from waste, thereby contributing to economic benefits. Moreover, copper slag-based electrocatalysis has the potential to significantly lower hydrogen production costs by eliminating reliance on expensive or rare semiconductor materials. Copper slag’s electrocatalytic properties make it particularly well suited for the hydrogen evolution reaction (HER) in seawater. Research indicates that its mesoporous structure enhances the efficiency of seawater splitting for hydrogen production. Additionally, the interaction between copper slag and seawater promotes the generation of electron–hole pairs, which drive the oxygen reduction reaction (ORR), complementing HER and establishing a balanced redox process [19,20,21]. The use of such water sources offers several advantages, as seawater accounts for 96.5% of the Earth’s total water resources and serves as a low-cost alternative. Furthermore, the presence of dissolved minerals such as chlorides, magnesium, and sodium can enhance the electrolysis process by increasing water’s electrical conductivity. This, in turn, reduces the energy required to split water into hydrogen and oxygen, improving the overall process efficiency [25].

The novelty of this study lies in the revalorisation of copper slag as an electrocatalytic material for sustainable hydrogen production in saline water electrolysis as a novel application. This approach aligns with the principles of circular economy and material recycling, transforming industrial waste into a valuable resource for clean technologies. By using copper slag as a cathode in electrolysis processes, the need for expensive noble metals is eliminated, significantly reducing production costs. Moreover, the use of seawater as the electrolyte marks a crucial step towards sustainability, as it enables the utilisation of an abundant and low-cost resource, avoiding the use of freshwater in arid regions. This innovative combination of a recycled material and a natural saline medium not only improves the technical and economic viability of electrolysis but also reduces environmental impact and promotes the generation of green hydrogen in coastal areas with high solar potential. This study considered a qualitative characterisation using X-ray diffraction (XRD), Raman spectroscopy, and scanning electron microscopy with energy-dispersive spectroscopy (SEM-EDS), along with electrochemical evaluations in 0.5 M NaCl and, seawater, comparing the electrochemical response when the copper slags are in contact with geothermal water (El Tatio from Atacama Desert); the research assesses its structural, compositional, and catalytic properties. The findings demonstrate copper slag’s high stability and efficiency in facilitating HER, particularly in saline environments, highlighting its potential as a cost-effective and sustainable material for green hydrogen production in coastal regions.

## 2. Materials and Methods

### 2.1. Copper Slag Preparation

Copper slag is a residue produced in a pyrometallurgical process to obtain copper concentrated from a concentrate of sulphide minerals, which contains materials such as FeO, SiO_2_, Al_2_O_3_, CaO, and Cu [14]. In the pyrometallurgical stage known as smelting, very high-temperature furnaces are used. As a consequence of the high temperatures, immiscible liquid phases are produced in this process: the copper-rich matte (sulphide) and the copper slag called oxide [7]. The Antofagasta region of Northern Chile is among the main copper-producing regions of Chile. There are two copper smelters in the Antofagasta region. In Northern Chile, there are copper deposits of current economic relevance, such as Chuquicamata (Calama) and Alto Norte (Antofagasta), both of which are in an operational phase. In addition, the presence of remnants of old smelters, dating back to the 18th century, has generated an extensive dispersion of copper-bearing slag throughout the desert of this region. This accumulation of historical metallurgical by-products represents a potential source of geochemical and archaeometallurgical analysis [26]. The copper slag sample used for the preparation of the electrode is from a smelter in the Antofagasta region. Copper slag powder was obtained by comminuting the material to increase the available surface area for chemical reactions. Initially, copper slag rocks were crushed into smaller fragments to facilitate further processing. These fragments were then pulverised into finer particles, ensuring uniformity in particle size. Finally, the material was sieved to achieve a particle size of −400# mesh, resulting in a fine copper slag powder with optimal characteristics for subsequent applications.

### 2.2. Preparation of Copper Slag Electrodes

**Figure 1:**
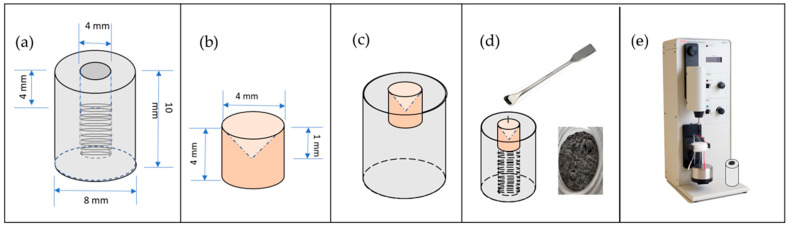
Construction of copper slag electrode. (**a**) PTFE cylinder, (**b**) concave bronze rod, (**c**) bronze rod inserted into PTFE cylinder, (**d**) copper slag powder paste deposited onto rod, (**e**) assembled electrode was integrated into RDE equipment.

The electrode onto which the copper slag powder is deposited consists of a PTFE cylinder measuring 8 mm in diameter and 10 mm in length, serving as a fixing device on the shaft of the rotating disc electrode cell. A concave bronze rod (4 mm in diameter and 4 mm in length) is inserted concentrically into this cylinder. In preparing the working electrode, a modified carbon paste was formulated by homogenising graphite powder (50% *w*/*w*) and solid paraffin (50% *w*/*w*). Modified carbon paste is used to prepare copper slag electrodes because of its wide availability, good conductivity, and ease of preparation [27]. This resulting paste was carefully deposited on a bronze rod with a concave configuration, designed to ensure efficient electrical conductivity with the rotating disc electrode (RDE) system. A quantity of copper slag powder is then applied and pressed to adhere to the surface. The assembled electrode was integrated into the RDE equipment for electrochemical analysis. To ensure the robustness and representativeness of the electrochemical data, the complete preparation and measurement procedure was replicated three times (see Figure 1).

### 2.3. Preparation of Saline Electrolyte

Saline solutions of 0.5 M NaCl were prepared using analytical-grade with ≥99.5% of purity of NaCl from Merck (Merck, Darmstadt, Germany) and deionised water. Seawater and geothermal water (El Tatio) were also used due to their high contents of ions such as chlorides, sodium, and magnesium, which act as natural electrolytes. These ions enhance the conductivity of the medium, making seawater suitable for electrochemical studies to evaluate charge transfer processes and redox reactions under conditions similar to real environmental scenarios [25]. Zhao et al. presented the chemical composition of seawater across different marine regions globally. Their research underscores that seawater primarily consists of dissolved ions, significantly determining its salinity and physicochemical properties. The average concentrations of the principal ions in seawater, measured in milligrams per litre (mg/L), are as follows: Cl^−^ (19.229 mg/L), Na^+^ (1.114 mg/L), Mg^2+^ (1.279 mg/L), Ca^2+^ (359 mg/L), K^+^ (340 mg/L), and SO_4_^2−^ (2.649 mg/L). El Tatio geothermal water plentiful in hot springs and geysers, located in the Andes in the region of Antofagasta, Northern Chile, 80 km from San Pedro de Atacama (22°20′ S, 68°01′ W), at an altitude of 4200 m a.s.l. It has a pH of 6 to 8 and a conductivity of ~20 mS/cm. Its discharged waters have high concentrations. The geothermal water (El Tatio) composition is as follows: Cl^−^ (6000–8000 mg/L), Na^+^ (>3500 mg/L), SO_4_^2−^ (<50 mg/L), Mg^2+^ (50–150 mg/L), Ca^2+^ (100–300 mg/L), and HCO_3_^−^ (200–500 mg/L) [28,29]. These values represent the general chemical composition of seawater worldwide, though minor variations can be observed depending on the oceanic region, depth, and specific environmental conditions [30,31]. Among these constituents, Cl^−^ and Na^+^ are the predominant electrolytes, playing a fundamental role in ion transport and enhancing the electrical conductivity of seawater.

### 2.4. Characterisation of Copper Slag Powder

#### 2.4.1. X-Ray Diffraction (XRD)

The XRD patterns of the copper slag powders were recorded on a Bruker Advance D8 diffractometer (Billerica, MA, USA). A Cu Kα1 (λ = 1.5406 Å) radiation source a Vertical Bragg-Brentano goniometer set at 40 kV and 30 mA and a scintillation detector were used. The XRD patterns were obtained in the 2θ range from 10° to 70°, with a step size and dwell time of 0.020° (2θ) and 3.0 s per step, respectively.

#### 2.4.2. Scanning Electron Microscopy (SEM-EDS)

General characterisation was performed using scanning electron microscopy (SEM) to reveal the morphological features of the microstructure. Surface analysis was carried out by SEM coupled with an energy-dispersive X-ray (EDX) spectrometer, employing a Zeiss EVA MA 10 microscope (Zeiss, Oberkochen, Germany). An accelerating voltage ranging from 5 kV to 15 kV was applied.

#### 2.4.3. Raman Spectroscopy

Raman spectra were obtained using a Raman Confocal Microscope, LabRAM Soleil, serial number: 1068, equipped with a 532 nm laser (gratings: 600 lines/nm) Horiba (Chelmsford, Essex, UK). LabSpec 6 and OriginPro 2023 (64-bit) (Copyright © 1991-2022 OriginLab Corporation, Northampton, MA, USA), were used to analyse the spectra. 

### 2.5. Electrochemical Studies of Copper Slag Powder

Electrochemical analyses were carried out using a BASi RDE-2 rotating electrode interface connected to an Epsilon potentiostat/galvanostat (Basi, West Lafayette, IN, USA). The analyses were performed separately by linear sweep voltammetry (LSV) in a conventional three-electrode cell setup, using copper slag powder as the working electrode, a platinum (Pt) wire as the counter electrode, and a silver/silver chloride (Ag/AgCl (4 M KCl sat.)) electrode as the reference. The experimental protocol for polarisation data was as follows: The cell was filled with the saline electrolyte and bubbled with air for approximately 15 min until an oxygen-saturated concentration was reached. The working electrode was then inserted into the cell, and linear sweep voltammetry was conducted within a potential range from −1200 to +200 mV/SHE at a scan rate of 2 mV/s. The rotation speed for the copper slag electrode was set to 1200 rpm.

### 2.6. Corrosion Studies for Copper Slag Powder

The experimental corrosion procedure was designed to examine the corrosion behaviour of partial electrochemical reactions on copper slag powder electrodes immersed in 0.5 M NaCl, seawater, and geothermal (El Tatio) solutions, focusing on the hydrogen evolution reaction (HER), oxygen reduction reaction (ORR), and copper slag oxidation reaction (CSOR). The temperature was maintained at 20 ± 0.5 °C using a water-jacketed cell that circulated water through a thermoelectric temperature control device. All experiments were repeated in triplicate. All potential measurements are referenced to the standard hydrogen electrode (SHE). The kinetic corrosion investigation was conducted using a non-linear fitting that was applied to experimental polarisation data [32]. The superposition model based on mixed potential theory was employed, incorporating both cathodic and anodic processes. The analysis focused on charge transfer, mass diffusion, and passivation mechanism controls, where the total current density can be expressed in Equation (1).

(1) $$i=i_{O_{2}}+i_{H_{2}}+i_{CS}$$

where i  represents the total current density, $i_{O_{2}}$, $i_{H_{2}}$, and $i_{CS}\;$ represent the partial reduction current densities for the oxygen reduction reaction (ORR) and hydrogen evolution reaction (HER), respectively, and $i_{CS}$ represents the partial oxidation current density for the copper slag oxidation reaction (CSOR). Although the experimental measurement of $i_{O_{2}},\;i_{H_{2}}$, and $i_{CS}$ is not directly feasible, their values can be deduced by considering the kinetic expressions for each reaction.

For this purpose, the partial reactions for HER and CSOR are modelled considering a charge transfer kinetic mechanism, while the ORR is described using a mixed charge-diffusion kinetic mechanism, according to the following expressions:

(2) $$i_{H_{2}}=i_{0,H_{2}}exp\left(-\frac{2.303\cdot \eta_{H_{2}}}{t_{H_{2}}}\right)$$

(3) $$i_{O_{2}}=i_{0,O_{2}}exp\left(-\frac{2.303\cdot \left(E-E_{eq,\;O_{2}}\right)}{t_{O_{2}}}\right)\cdot {\left(1-\frac{i_{O_{2}}}{i_{l,O_{2}}}\right)}^{0.5}$$

(4) $$i_{CS}=i_{0,CS}exp\left(\frac{2.303\cdot \eta_{CS}}{t_{CS}}\right)$$

where $i_{0,H_{2}},\;i_{0,O_{2}},\;i_{0,CS}$, are the exchange current densities for HER, ORR, and CSOR, respectively. $i_{l,O_{2}}\;$ represents the limiting current density for ORR. The overpotentials for HER, ORR, and CSOR are expressed as son $\eta_{H_{2}}=E-E_{eq\;H_{2}},\;\eta_{O_{2}}=E-E_{eq\;O_{2}}$ and $\eta_{CS}=E-E_{eq\;CS}$, respectively. The equilibrium potentials for HER, ORR, and CSOR are denoted as $E_{eq\;H_{2}},\;E_{eq\;O_{2}\;}$, and $E_{eq\;CS}$, respectively. The cathodic Tafel slopes for HER and ORR are $t_{H_{2}}$ and $t_{O_{2}}$, respectively, while $t_{CS}\;$ represents the anodic Tafel slope for CSOR.

The exchange current densities $\left(i_{o,\;j}\right)$, have been determined using the Butler–Volmer equation at the equilibrium potential for each partial reaction involved in the electroanalysis. To achieve this, we have used electrochemical parameters obtained from the non-linear fitting of experimental data. The equation can be simplified as follows:

(5) $$i_{o,j}=a\;exp(b\cdot E_{eq,j})$$

where a is base current (i) and b is sensitivity to potential, are the fitted parameters derived from Equation (1).

## 3. Results

*Structural and Morphological Analysis of Copper Slag*

**Figure 2:**
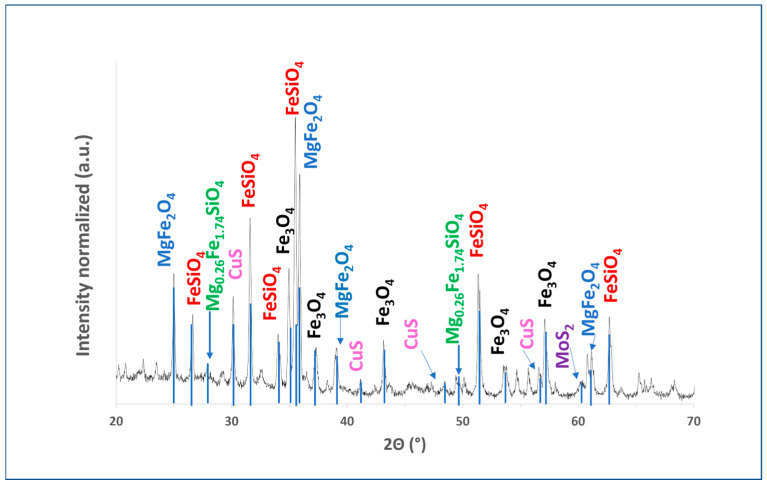
XRD diffractogram of copper slag powder showing peaks for fayalite and magnesioferrite. Data were collected using Bruker Advance D8 diffractometer (Cu Kα1, λ = 1.5406 Å) with 2θ range of 10–70°, 0.020° step size, and 3.0 s dwell time per step.

Figure 2 shows the X-ray diffraction (XRD) spectra. The figure illustrates the copper slag dust patterns, showing that the main phases present in this material are fayalite (Fe_2_SiO_4_) and magnetite (Fe_3_O_4_).

**Figure 3:**
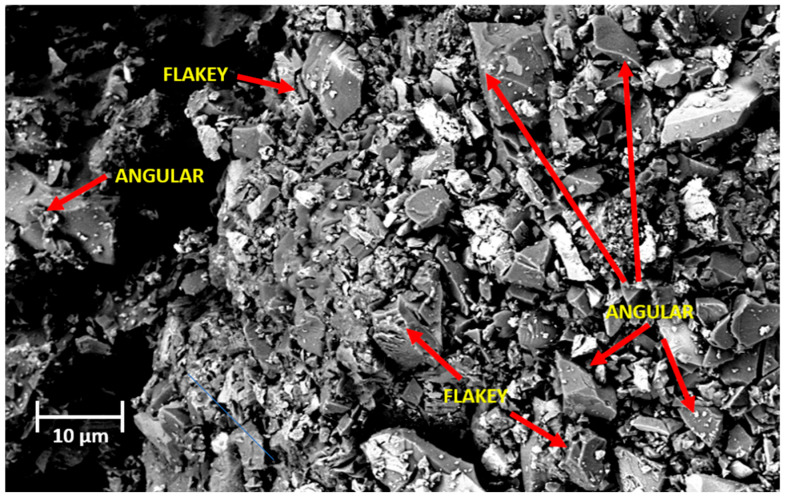
SEM image of copper slag powder before exposure to saline solutions, acquired under BSD1 mode at 1.74 KX magnification, 5.00 kV accelerating voltage, 7.2 mm working distance, and 10 µm scale.

Figure 3 shows an image of copper slag powder before contact with salt solutions through the scanning electron microscope (SEM), the signal is under BSD1, magnitude 1.74 KX, ETH 5.00 Kv, WD 7.2 mm, and at 10 µm. It was observed that the copper slag powder shows a diversity of crystalline phases. Mostly angular and flaky particles are observed. In addition, the copper slag powder shows the presence of elements of higher molar mass due to its brightness.

**Figure 4:**
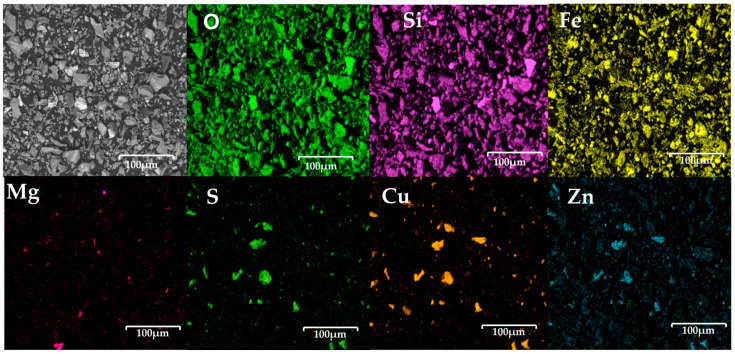
Point analysis of copper slag powder electrodes before contact with seawater, showing distribution of key elements such as oxygen, silicon, iron, and magnesium, as well as presence of residual sulphur, copper, and zinc.

The point analysis, presented in Figure 4, reveals, in general terms, that iron is present in 12.1 wt%, oxygen represents 28.2 wt%, and silicon accounts for 7.3 wt%. This suggests that the copper slag powders have a high iron oxide content.

**Figure 5:**
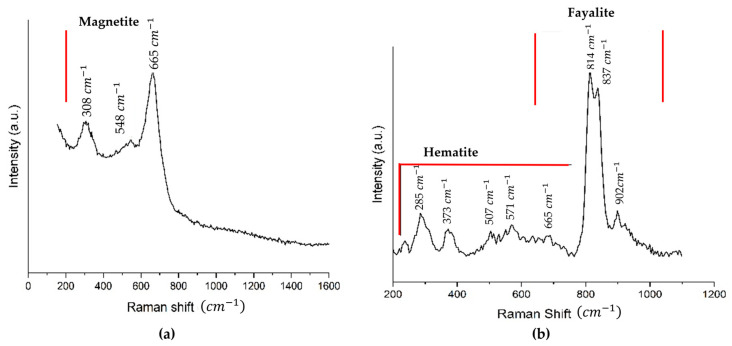
(**a**) The Raman spectra of the dark areas of the copper slag sample, (**b**) In the lightest part of the sample, the olivine-type fayalite can be seen. The red line indicates the zone where are representative signal of each component (magnetite, hematite and fayalite).

The Raman spectrum analysis shows the presence of magnetite (Fe_3_O_4_), hematite (Fe_2_O_3_), and olivine (fayalite type (Mg,Fe)_2_SiO_4_). In Figure 5a, the Raman spectrum of a darker zone of the sample is shown, which coincides with the main presence of magnetite. The analysis of the Raman spectrum of the copper slag presented in Figure 5b, which shows the spectrum of a lighter area, presents mostly fayalite in the form of olivine.

**Figure 6:**
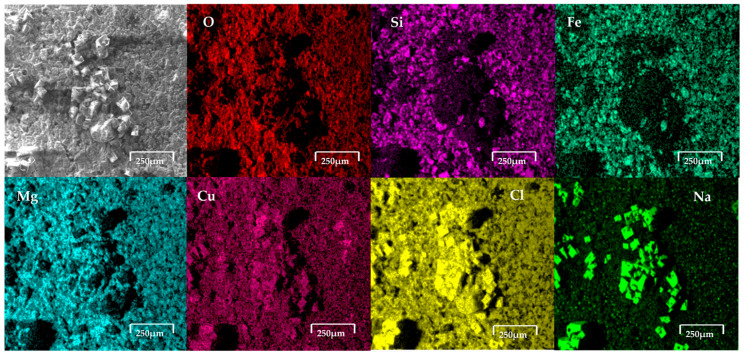
SEM-EDS analysis of copper slag powder electrodes after contact with saline. It clearly shows presence of cubic crystals on electrode surface. These crystals, according to EDS, correspond to NaCl.

Figure 6 shows the copper slag modified carbon paste electrode after the oxidation process in 0.5 M NaCl. The results revealed the presence of cubic crystals on the electrode. Elemental analysis via EDS, as shown in Figure 6, reveals the presence of cubic crystals of NaCl, indicating the contribution of the NaCl solution to the observed composition.

**Figure 7:**
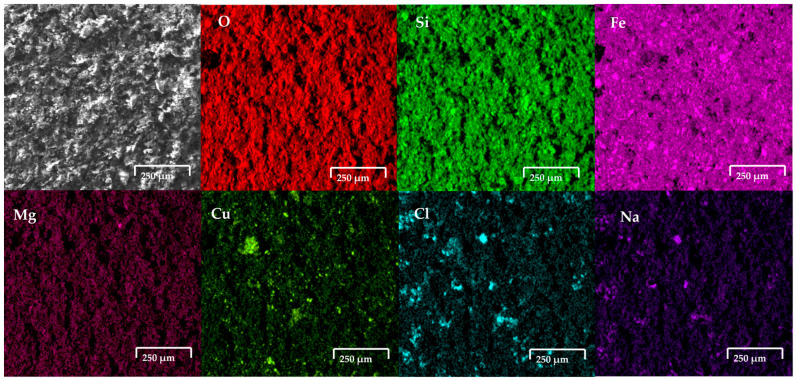
SEM-EDS analysis of copper slag powder electrodes after contact with seawater.

Figure 7 shows the modified carbon paste electrode with copper slag after the oxidation process in seawater. The EDS analysis confirmed the presence of elements characteristic of copper slag powder, including Cu (5.2 wt%), Fe (39.9 wt%), Si (13.4 wt%), and O (29.2 wt%), along with the incorporation of ions from the saline medium, such as ions Cl^−^ and Na^+^, respectively.

**Figure 8:**
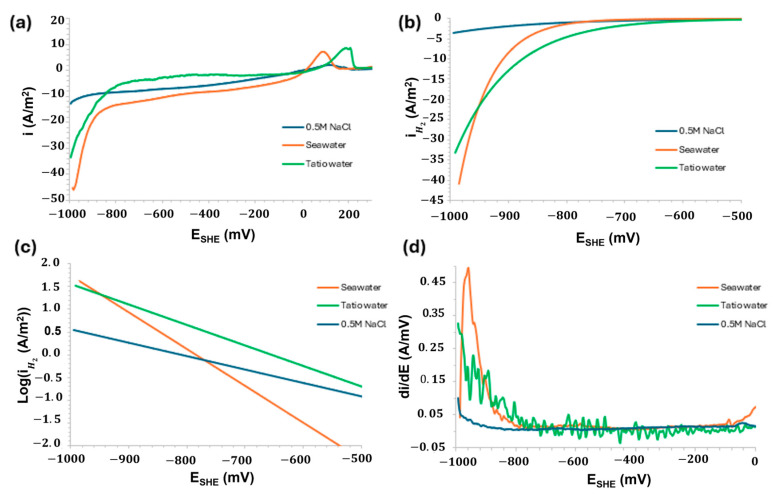
Electrochemical performance of 0.5 M NaCl, seawater, and geothermal water (El Tatio, Atacama Desert). (**a**) Lineal voltammetry (LSV), (**b**) H_2_ evolution, (**c**) H_2_ Tafel slope, and (**d**) behaviour of first derivative (di/dE).

Figure 8 provides important insights into the electrochemical kinetic performance of copper slag powder during the sub-cathodic process in contact with 0.5 M NaCl, seawater, and geothermal water from El Tatio (Atacama Desert), respectively.

## 4. Discussion

Previous studies, such as that by Allibai et al. [33], focus on the development of electrochemical sensors based on nanocomposites like Fe_2_O_3_ and copper hexacyanoferrate (CuHCF) for detecting specific analytes such as hydroxylamine in aqueous media. This manuscript proposes a broader and more impactful application in the context of the energy transition: the direct use of copper slag as an electrocatalytic material for the hydrogen evolution reaction (HER) in seawater electrolysis. In contrast, the work of Li et al. [34]. centres on the chemical and mineralogical characterisation of copper slag, evaluating its environmental hazards through leaching and sequential extraction tests, with an emphasis on the recovery of metals such as iron, copper, and lead. The current study goes a step further by actively valorising copper slag through its direct integration into electrolysis cells, without requiring prior mineral separation or concentration processes. This strategy not only reduces costs and material waste but also aligns with the principles of circular economy and water sustainability—particularly significant in one of the driest regions on Earth. Taken together, this research offers an innovative, multifunctional, and environmentally responsible approach the utilisation of industrial waste, paving the way for the sustainable and decentralised production of green hydrogen [33,34].

Iron oxides and sulphides—such as magnetite (Fe_3_O_4_), haematite (Fe_2_O_3_), and pyrite (FeS_2_)—have emerged as promising electrocatalytic materials owing to their low toxicity, high abundance, low cost, and favourable chemical stability. These compounds possess semiconducting properties and well-defined crystalline structures that facilitate charge transfer and the adsorption of reactive species during electrochemical processes such as the hydrogen evolution reaction (HER). Specifically, magnetite and haematite can serve as active sites for water molecule dissociation, thereby promoting the generation of molecular hydrogen. Pyrite and other iron sulphides have also demonstrated notable electrocatalytic behaviour, particularly when incorporated into hybrid materials or supported on conductive substrates. Recent studies have shown that the modification of electrodes with Fe_2_O_3_ nanoparticles significantly enhances HER performance by increasing the electroactive surface area and improving charge transfer kinetics. Similarly, synergistic effects arising from combinations of metal sulphides and mixed oxides have been shown to enhance catalytic activity, even under saline conditions. Given their natural abundance and compatibility with waste-derived systems such as copper slag, these materials are well-positioned to contribute to the development of green hydrogen technologies within the framework of sustainable energy transition [20,33,35].

Research into pyrite (FeS_2_) and its derivatives as electrocatalysts has garnered growing attention due to their natural abundance, low cost, and advantageous structural characteristics for key electrochemical processes such as the oxygen evolution reaction (OER) and hydrogen evolution reaction (HER). Studies have shown that natural pyrite, when supported on conductive substrates such as nickel foam, exhibits exceptionally high catalytic activity, often comparable to that of IrO_2_, highlighting the critical role of synergistic interactions between the catalyst and its support. In parallel, various strategies have been explored to enhance catalytic efficiency and address limitations such as weak proton (H^+^) adsorption commonly found in certain iron sulphides. These strategies include heteroatomic alloying (e.g., Ni doping in FeS_2_), nanostructuring, and fine-tuning of crystallographic parameters such as crystallite size and lattice strain. Such modifications have been shown to significantly improve the bifunctional activity of pyrite in alkaline media, with performance approaching that of commercial catalysts like RuO_2_–Pt/C. Beyond OER and HER, recent studies have revealed the potential of FeS_2_ as a photocatalyst for electrochemical nitrogen reduction, further underscoring its functional versatility. Structural variants such as cobalt pyrite (CoS_2_) and hybrid systems like CoPS supported on carbon nanotubes have also demonstrated low overpotentials and high current densities. These findings emphasise that controlled synthesis, targeted elemental substitution (e.g., P or Ni doping), and integration with conductive frameworks are key strategies for enhancing electrocatalytic performance. Taken together, these advances reinforce the viability of pyrite and its derivatives as sustainable and efficient alternatives to noble metal-based catalysts, consolidating their relevance in the transition towards clean, affordable, and decentralised energy conversion technologies [36,37,38,39,40,41,42].

During the cathodic subprocess of electrolysis in seawater, in addition to the hydrogen evolution reaction (HER), critical operational issues may arise that affect the system’s efficiency and durability. One of the most significant is the formation of inorganic scaling, caused by the precipitation of poorly soluble salts such as calcium carbonate (CaCO_3_), magnesium hydroxide (Mg(OH)_2_), and calcium sulphate (CaSO_4_), induced by the local increase in pH at the cathode interface. These deposits reduce the active surface area of the electrode, increase charge transfer resistance, and may obstruct gas release channels, thereby lowering the efficiency of hydrogen production. In parallel, seawater contains a significant load of microorganisms, nutrients, and organic matter, which promote the formation of biofouling layers on the cathode surface [43,44,45]. These biological films can act as diffusional barriers, alter the surface microstructure of the electrode, and facilitate localised corrosion, particularly in materials with chemical heterogeneity such as copper slags. The simultaneous presence of mineral scaling and biofouling presents major challenges for the continuous and stable operation of the system [46]. Therefore, assessing the resistance of residual materials such as copper slag to these phenomena under real conditions (i.e., untreated seawater) is essential to determine their practical viability as cathodes in low-cost and sustainable electrolysis technologies.

It is true that materials such as platinum (Pt) exhibit exceptional catalytic activity and are the benchmark for the hydrogen evolution reaction (HER) under ideal conditions [47,48]. However, the aim of this study is not to compete directly with noble metals or advanced materials synthesised under controlled laboratory conditions, but rather to evaluate the potential of copper slag as a low-cost and functional electrocatalyst under real-world conditions, using untreated seawater as the electrolyte. From this perspective, although the current density achieved by copper slag in 0.5 M NaCl is lower than that reported for commercial catalysts, its electrochemical stability and activity in complex media such as seawater and geothermal water (El Tatio) represent a significant step forward towards practical, sustainable, and low-cost applications, particularly in arid regions such as Northern Chile, where this type of waste is readily available and access to freshwater is limited.

Moreover, there is growing interest in the use of natural or waste-derived materials as electrodes. For example, the use of natural pyrite (FeS_2_) has been reported with promising results for OER on nickel foam, and strategies such as doping and nanostructuring have been explored to improve the performance of metal sulphides like CoS_2_ and MoS_2_, though with greater synthetic complexity and associated costs [49]. In this context, copper slag offers a direct alternative without the need for prior modification processes, making it easier to scale up and integrate into distributed hydrogen generation systems. We acknowledge the importance of contextualising our results in comparison with other electrocatalysts. In future versions of the manuscript, a comparative table will be included featuring representative materials such as Pt/C, MoS_2_, CoP, and mixed oxides, considering key parameters such as overpotential at 10 mA cm^−2^, Tafel slope, stability, and cost per unit of active area. This comparison will help to position copper slag more clearly as a viable alternative in decentralised and environmentally responsible electrolysis technologies [34,50,51,52,53].

Figure 2 illustrates patterns of copper slag dust, showing that the main phases present in this material are fayalite (Fe_2_SiO_4_) and magnetite (Fe_3_O_4_). According to Li et al., it is possible to identify complex silicates and other residual metallic elements in addition to these main phases [34]. Additionally, other phases observed in the copper slag dust sample include magnesioferrite (MgFe_2_O_4_), molybdenite (MoS_2_), and, to a lesser extent, the crystalline phase of covellite (CuS). Furthermore, taking into account the known origin of the sample, it can be stated that amorphous phases corresponding to mineraloids were identified. Furthermore, previous studies suggest that XRD may not detect certain residual metallic elements due to their low concentration or because they are dispersed within the copper slag [9,26,34,35,54,55].

The point analysis, presented in Figure 4, reveals, in general terms, that iron is present in 12.1 wt%, oxygen represents 28.2 wt%, and silicon accounts for 7.3 wt%. This suggests that the copper slag powders have a high iron oxide content. Additionally, 4.0% and 0.5 wt% were detected, indicating that a small amount of copper sulphide still remains in the slag powders. Given this composition, the copper slag exhibits significant potential for electrochemical applications, particularly due to its iron oxide content. In this regard, Allibai et al. demonstrated that modifying a wax-impregnated graphite electrode with iron oxide (Fe_2_O_3_) nanoparticles enhances the electron transfer kinetics and electrocatalytic activity while increasing the surface area of the electrode. This results in improved anodic responses for electrochemical sensing, specifically for hydroxylamine detection [33]. Considering the high concentration of iron oxide in the copper slag, it may be a promising candidate for similar electrochemical applications, where such enhancements could be beneficial.

The Raman spectrum analysis shows the presence of magnetite (Fe_3_O_4_), hematite (Fe_2_O_3_), olivine ((Mg, Fe)_2_SiO_4_), and fayalite (Fe_2_SiO_4_). In Figure 5a, the Raman spectrum of a darker zone of the sample is shown, which coincides with the main presence of magnetite. This reported band indicates three signals, two weak bands at ~308 1/cm (Eg) and ~548 1/cm (T2g) and one prominent band at ~665 1/cm. Das et al. indicate that the prominent bands of magnetite, whose broadband range for magnetite is ~661 to ~676 1/cm (A1g), are consistent with other authors [56]. The analysis of the Raman spectrum of the copper slag presented in Figure 5b shows the spectrum of a lighter area, which presents mostly fayalite in the form of olivine, showing three characteristic signals at ~813, ~837, and ~902 1/cm, respectively. Kuebler et al. observed that the positions of the olivine doublet peaks vary with composition, from olivine to forsterite [57]. Olivine has a characteristic signal as a doublet around 837–815 1/cm for symmetric Si-O stretching band and has one symmetric Si-O stretching band between 825 and 808 1/cm. According to the XRD pattern, this corresponds mainly to fayalite with some complement of forsterite with a composition containing magnesium (Mg) below 0.26 and iron (Fe) above 1.74. Hematite may have appeared by oxidation of magnetite upon interaction with the laser [58,59]. This indicates that the sample analysed belongs to the olivine solid solution series, with a predominance of its iron-rich phase [60].

The electrodes, composed of copper slag powder and carbon wax, were immersed separately in seawater and an aqueous solution with a high salt concentration and subjected to electrochemical analysis to assess their response to corrosion and other electrochemical processes. Following this procedure, the electrodes were dried and prepared for characterisation using SEM-EDS. The SEM technique was employed to investigate morphological alterations on the material surface, while EDS analysis enabled the determination of its surface chemical composition. The images obtained by SEM revealed modifications in the surface morphology of the electrode, characterised by the presence of a porous structure and crystalline formations on the surface. This suggests the occurrence of selective dissolution processes and the precipitation of compounds within the saline medium. Figure 6 shows the copper slag modified carbon paste electrode after the oxidation process in 0.5 M NaCl. The results revealed the presence of cubic crystals on the electrode. From EDS analysis, as presented in the figure, the elemental analysis indicates that the formation of cubic crystals is NaCl, which comes from the NaCl solution. This indicates that there may be NaCl crystals and ions present in seawater (no presence of CaCO_3_, Mg(OH)_2_ and CaSO_4_ was detected). This suggests that the copper slag dusts absorbed salts on their surface. Figure 7 shows the modified carbon paste electrode with copper slag after the oxidation process in seawater. The EDS analysis confirmed the presence of elements characteristic of copper slag powder, including Cu (5.2 wt%), Fe (39.9 wt%), Si (13.4 wt%), and O (29.2 wt%), along with the incorporation of ions from the saline medium, such as Cl^−^ and Na^+^. The detection of these elements indicates the formation of corrosion products and the deposition of salts on the electrode surface. The chemical composition determined by EDS following exposure to seawater was 2.9 wt% Na, 3.3 wt% Al, 1.5 wt% Cl, 1.2 wt% Ca, 1.4 wt% K, 0.7 wt% Mg, and 2.3 wt% Mo.

The kinetic study was performed by applying a non-linear fit to the experimental polarisation data, based on the superposition model and mixed potential theory. This theoretical framework assumes that both anodic and cathodic reactions are governed by charge transfer, mass diffusion, and the control of oxidation mechanisms. Figure 8 provides key insights into the electrochemical and kinetic behaviour of the copper slag powder electrode during the cathodic subprocess, as a function of the electrolyte used (0.5 M NaCl, seawater, and El Tatio geothermal water). Figure 8a presents the polarisation curves, revealing a clear cathodic response. Among the tested electrolytes, seawater exhibited the highest cathodic current, followed by geothermal water and, lastly, 0.5 M NaCl. This trend suggests that natural electrolytes possess higher conductivity, likely due to their complex ionic composition and enhanced oxygen availability—factors that favour the oxygen reduction reaction (ORR). These findings are consistent with those reported by Santos et al. and Tong et al., who observed improved cathodic activity in natural saline media, attributed to their greater ionic complexity [61,62]. The negative potential region (Figure 8b) enables a kinetic-focused analysis. In this region, seawater exhibits a steeper slope, indicating a higher exchange current density within the same potential range. The distinct Tafel slopes observed for the three electrolytes (Figure 8c) suggest that the HER mechanism on the copper slag surface is influenced by the specific composition of each electrolyte. Notably, the Tafel slope for 0.5 M NaCl is higher than those for seawater and El Tatio geothermal water, implying slower charge transfer kinetics in the NaCl solution. The differential derivative analysis (di/dE, Figure 8d) reveals abrupt changes in the reaction mechanisms. Peaks observed in seawater and geothermal water around −950 mV may be attributed to redox-active components within the electrolyte. In contrast, the curve for 0.5 M NaCl exhibits a more uniform profile, reflecting a more stable but less electrochemically active system.

The specific behaviour around 0 V/SHE, corresponding to seawater, is not as pronounced in 0.5 M NaCl. It can be attributed to the transition of the ORR mechanism, dissolution of passive layers, or the reduction in residual species. Due to the characteristic of the electropotential, which contains chloride ions and is in materials such as copper slag, which could have traces of Cu or metal sulphides that are reduced around 0 V vs. SHE, generating these additional anodic peaks. Grimm et al. demonstrated this effect in copper materials using saline electrolytes [63].

The comparative electrochemical and kinetic parameters of copper slag powder are summarised in Table 1. Copper slag exhibits greater activity for both the oxygen reduction reaction (ORR) and the hydrogen evolution reaction (HER) in seawater. According to Kaiser et al., copper alloys in saline environments display reduced corrosion resistance, which may contribute to the observed behaviour. The highest exchange current density was recorded in geothermal water, suggesting that the heterogeneous surface of the copper slag enhances its catalytic activity for the ORR under these conditions [64]. Studies by Yaro et al. and Khadom et al. indicate that corrosion kinetics are influenced by both the chemical composition of the electrolyte and the nature of the electrode material. The corrosion analysis further revealed that, although the corrosion potential $\left(E_{corr}\right)$ shifted towards more positive values and the corrosion current density $\left(i_{corr}\right)$ increased in seawater, this electrolyte’s composition accelerates corrosion in copper slag [65,66]. In contrast, Metikos-Hokovic et al. emphasise that both flow dynamics and surface composition affect passive film formation, which supports the lower corrosion rate observed in geothermal water, evidenced by its more positive $\left(E_{corr}\right)$ and reduced $\left(i_{corr}\right)$ values [67].

## 5. Conclusions

This study demonstrates the feasibility of using untreated copper slag as a low-cost and sustainable electrocatalyst for hydrogen production through saline water electrolysis. Through comprehensive physicochemical and electrochemical characterisation—including XRD, SEM-EDS, Raman spectroscopy, and kinetic modelling—we establish that copper slag exhibits favourable surface properties, high electrochemical stability, and catalytic activity toward the hydrogen evolution reaction (HER), particularly in seawater and geothermal brine environments. These findings underscore the potential of this abundant industrial by-product as a functional electrode material that bypasses the need for expensive noble metals.

Notably, the material’s performance in real electrolytes such as seawater and El Tatio geothermal water validates its robustness under complex, high-salinity conditions. The electrochemical response confirms that copper slag not only withstands corrosive media but also facilitates effective charge transfer, positioning it as a viable option for decentralised, low-cost hydrogen generation systems in arid coastal regions like Northern Chile.

This approach aligns with circular economy principles, offering a dual benefit: waste valorisation and green hydrogen production. Future work should focus on the long-term operational stability of copper slag electrodes under continuous use, the mitigation of scaling and biofouling effects, and the integration of this material into pilot-scale electrolysis systems. Such efforts will be essential to assess its commercial scalability and full environmental impact in sustainable energy applications.

## Figures and Tables

**Table 1:** Electrochemical parameters and kinetic properties of copper slags in contact with salt solutions.

Parameters	0.5 M NaCl	Seawater	Geothermal Water (El Tatio)
$i_{0,CS}$, A m^−2^	0.1066	0.0021	0.0011
$t_{CS}$, mV dec^−1^	338	140	157
$i_{0,O_{2}}$, A m^−2^	0.097	0.380	0.0186
$t_{O_{2}}$, mV dec^−1^	−587	−764	−417
$i_{l,O_{2}}$, A m^−2^	−7.8	−11.9	−2.1
$i_{0,H_{2}}$, A m^−2^	−0.383	−84.896	−0.132
$t_{H_{2}}$, mV dec^−1^	−263	−126	−221.4
$E_{corr}$, mV	−1.124	12.539	30
$i_{corr}$, A m^−2^	2.117	3.668	1.02

## Data Availability

The original contributions presented in this study are included in the article. Further inquiries can be directed to the corresponding authors.
